# Supplementary material for: Self-Assembled Nanoparticles from Cationic Dipeptides and D-π-A Chromophores for Near-Infrared Photothermal Therapy
Source: Int J Mol Sci. 2025 Nov 20;26(22):11235. doi: 10.3390/ijms262211235 (PMC12653464; doi:10.3390/ijms262211235)
Supplement: Supplementary file 1 [file ijms-26-11235-s001.zip › ijms-3966673-supplementary.pdf]

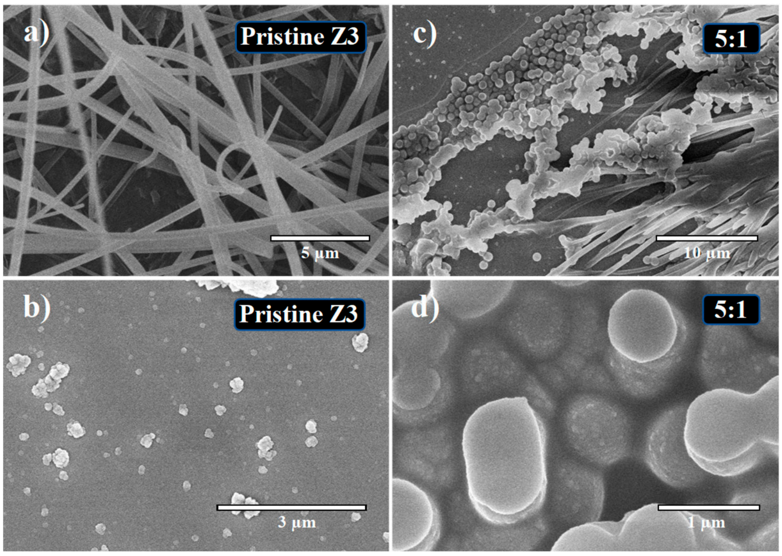

Figure S1. SEM images showing the self-assembled structures of pristine Z3 and CDPNCs-Z3 (CDP: Z3 mass ratio = 5:1) under high-concentration (a,c) and diluted (b,d) conditions.

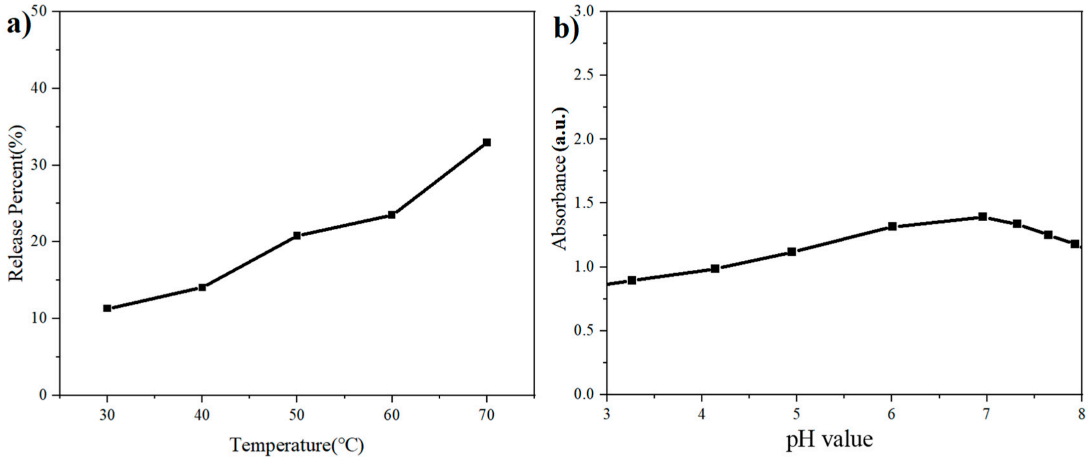

Figure S2. Temperature- and pH-responsive release behavior of the CDPNCs-Z3 composite nanoparticles. (a) Release profiles based on the absorbance at 710 nm under different temperatures. (b) Turbidity curve of the CDPNCs-Z3 suspension as a function of pH.
